# Necrotizing Bacterial Myositis as the Initial Presentation of Severe Aplastic Anaemia

**DOI:** 10.1155/2021/8276937

**Published:** 2021-12-21

**Authors:** Synne Dragesund Rørvik, Kristoffer Stange Larsen, Lars Helgeland, Håvard Dale, Birgitta Ivarsen, Øystein Bruserud, Tor Henrik Anderson Tvedt

**Affiliations:** ^1^Department of Medicine, Haukeland University Hospital, Bergen, Norway; ^2^Department of Medicine, Haraldsplass Deaconess Hospital, Bergen, Norway; ^3^Department of Pathology, Haukeland University Hospital, Bergen, Norway; ^4^Department of Clinical Medicine (K1), University of Bergen, Bergen, Norway; ^5^Department of Orthopaedic Surgery, Haukeland University Hospital, Bergen, Norway; ^6^Department of Plastic Surgery, Haukeland University Hospital, Bergen, Norway; ^7^Department of Haematology, Oslo University Hospital, Rikshospitalet, Oslo 0424, Norway

## Abstract

**Introduction:**

Necrotizing soft tissue infections are rapidly progressing infections associated with severe inflammation and cytokine release. Early recognition and surgical intervention are key factors to secure survival. The current case presents a patient with multifocal necrotizing soft tissue infection as the initial presentation of severe aplastic anaemia. *Case Presentation*. A man in his fifties was admitted with septic shock with multiorgan failure and severe pancytopenia, after two days of malaise with high fever and right flank pain. The diagnosis streptococcal necrotizing myositis was significantly delayed due to atypical clinical findings. After initial surgical exploration, the decision was made to defer from surgical debridement due to extensive involvement of several muscle groups, grave pancytopenia, and suspected dismal prognosis. Surprisingly, the patient stabilized after antibiotics and intensive care treatment. Based on severe pancytopenia and hypocellular bone marrow, with no evidence of other bone marrow disorders, the patient was diagnosed with aplastic anaemia. Treatment for aplastic anaemia with antithymocyte globulin, cyclosporine, and eltrombopaq was started, and 2 months later, a partial haematological recovery was observed. The patient could be discharged from hospital without antibiotic treatment.

**Conclusions:**

This case illustrates the crucial role of a multidisciplinary approach on admission and further during the clinical course. Clinical improvement despite severe neutropenia and stabilization during immunosuppressive therapy suggest that immunological factors modulate clinical course in necrotizing soft tissue infections.

## 1. Introduction

Necrotizing soft tissue infections (NSTIs) are characterized by significant inflammation, multiorgan failure, morbidity, and mortality [1]. A dramatic increase in mortality is seen if surgery is delayed or surgical debridement is inadequate [2, 3]. Furthermore, the initial inflammatory response contributes to the early mortality as well as the prolonged recovery and late mortality [4, 5]. Although several studies have demonstrated that age, presence of chronic diseases, steroid use, previous blunt trauma, and a lower body mass index are risk factors for NSTIs [6, 7], the underlying pathophysiological mechanisms are poorly understood. However, experimental data suggest that bacterial pyrogenic exotoxin-induced immune activation contributes to muscle inflammation as well as compromised cellular and humoral immunity [8]. In the current article, we describe a patient with NSTI in several muscle groups as the initial presentation of aplastic anaemia (AA) with severe neutropenia. Despite a significant diagnostic delay, the patient improved gradually without extensive surgical intervention and response to immunosuppressive therapy did not exuberate the muscle disease.

## 2. Case Presentation

A 50-year-old male was admitted after two days of malaise, high fever, and right flank pain. His medical history included bilateral Calve-Legg-Perthes disease treated with left side total hip arthroplasty, gout, and hypothyroidism for which he received allopurinol, diclofenac, and levothyroxine, respectively. He had no known prior haematological diseases and had a normal haemoglobin level and peripheral white blood cell counts at his last routine examination, 18 months prior to admission. He denied having fever, with the exception of the days prior to admission, neither the skin nor mucosal bleeding, weight loss, or night sweats.

He was severely hypotensive at admission with a systolic blood pressure of 50 mmHg; he appeared drowsy and lethargic but responded adequately to questions and commands. Clinical examination revealed a bluish red discoloration on the right flank without additional prominent findings with no obvious signs of previous penetrating or blunt trauma. Arterial blood gas analysis showed a compensated metabolic acidosis with increased lactate level 4.2 mmol/L (normal level 0.9–1.7 mmol/L). Empiric antibiotic treatment with penicillin G plus gentamicin together with fluid resuscitation and noradrenaline-infusion was immediately initiated. The blood pressure subsequently stabilized at 100/60 mmHg. Initial blood sample testing showed the following results:

- A significant acute-phase response with a C-reactive protein (CRP) level of 592 mg/L (normal level <5 mg/L), procalcitonin 56.2 *μ*g/L (<0.1 *μ*g/L), and fibrinogen 8.6 g/L (1.9–4.0 g/L)
- Severe pancytopenia with total peripheral blood leucocytes counts below 0.5 × 10^9^/L (3.5–11.0 × 10^9^/L), neutrophils 0.1 × 10^9^/L (1.7–8.2 × 10^9^/L), haemoglobin 6.1 g/dL (13.4–17.0 g/dL), reticulocytes 0.01 × 10^12^/L (0.03–0.1 × 10^12^/L), and thrombocytes 3 × 10^9^/L (145–348 × 10^9^/L). Activated partial thromboplastin time and international normalized ratio were both within normal reference values.
- Severe renal failure with creatinine 430 *μ*mol/L (60–105 *μ*mol/L)
- Evidence of myonecrosis with a myoglobin level of 6,486 *μ*mol/L (<70 *μ*mol/L), creatinine kinase level of 10,430 U/L (40–80 U/L), and lactate dehydrogenase 340 U/L (105–205 U/L)

A full-body computed tomography (CT) showed a subcutaneous haematoma of the right flank, but without signs of thickened fascia, gas in the soft tissue, or signs of injury to other organs or bone structures. The liver and spleen appeared normal. At this time point, the patient was transferred to the intensive care unit (ICU) at our university hospital, and progression of the skin lesion was noted at admission. The lesion now included a well-demarcated erythema involving the entire right half of the torso, abdomen, and gluteal region, but neither bulla nor skin necrosis was present. There was no crepitus formation, fluctuations, or pain on touch. The clinical picture was considered as possible deep tissue infection, but a watchful waiting strategy was chosen because of the high risk associated with major surgery in patients with severe pancytopenia and the absence of typical NSTI findings. Antibiotic treatment was changed to meropenem plus clindamycin. Ultrasound did not reveal presence of abscesses or gas. Gram stain of fluids aspirated from the affected areas was negative for bacteria, and cytological examination did not indicate that the subcutaneous fluid originated from a haematoma, as indicated by CT. Blood cultures at admission and during the subsequent clinical course were all negative.

Further diagnostic work-up was done to evaluate the cause of the bone marrow failure. The bone marrow smear showed a significantly hypocellular, almost acellular marrow. No megakaryocytes were present. There was no increase in blast cells, and hemophagocytosis was not present. The smear was dominated by mature lymphocytes and monocytes, and there were no clear signs of myelodysplasia. Immunophenotyping of peripheral blood and bone marrow aspirate (analysed according to standard EuroFlow protocol) showed no presence of lymphoma, leukaemia, myelodysplastic syndrome, or paroxysmal nocturnal haemoglobinuria. The levels of folic acid, cobalamin, zinc, lead, copper, selenium, mercury, cadmium, and metabolic hormones were within normal range. Serology of parvovirus, human immunodeficiency virus (HIV), hepatitis B and C, Epstein-Barr virus, and cytomegalovirus was negative. Based on initial findings, the diagnosis of severe aplastic anaemia was suspected, and treatment with granulocyte colony-stimulating factor (G-CSF) was therefore initiated while waiting for the results from cytogenetic bone marrow examination and bone biopsy.

**Figure 1:**
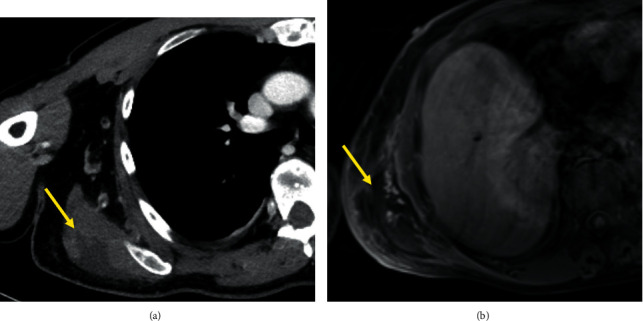
(a) Coronal images from a contrast CT of the thorax showing right latissimus dorsi muscle suggestive of deep tissue infection with possible muscle necrosis. (b) Coronal images from a contrast MRI of the thorax showing myonecrosis of the right latissimus dorsi muscle.

**Figure 2:**
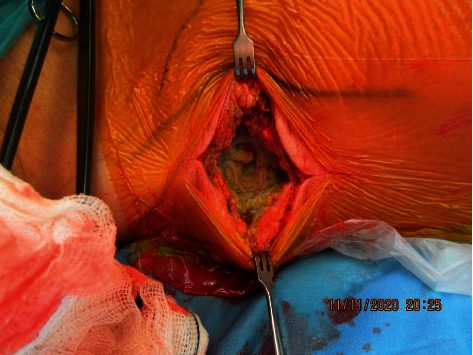
Intraoperative images of the right latissimus dorsi muscle. Thickened and greenish fascia with underlying necrotic muscle.

Over the next 48 hours, vasoactive agents could be weaned off, lactate levels normalized, renal function improved, and myoglobin and creatinine kinase levels decreased significantly. The patient could be discharged from the ICU. However, the erythema on the right flank/hip increased and became more painful. A repeated CT of the right latissimus dorsi muscle (Figure 1(a)) suggested deep tissue infection with muscle necrosis, and a magnetic resonance tomography imaging (MRI) was also consistent with myonecrosis (Figures 1(b)). It was therefore decided to proceed to explorative surgery. Upon surgical exploration, the fascia of the right latissimus dorsi muscle was found to be greenish and thickened, and the underlying muscle was necrotic with an absence of bleeding and contractility upon stimulation (Figure 2), but without smell or gas release. The findings were regarded as diagnostic for necrotizing bacterial myositis. A complete debridement of the necrotic muscle would have resulted in a wound area reaching from the right shoulder through the entire dorsal area of the chest, abdomen, and gluteal region, and despite the severe bone marrow failure, the clinical condition was at this time stable with a decrease of CRP levels. Further surgical debridement was therefore deferred. Perioperative tissue and fluid samples were obtained for microbiological and histopathological analyses. Microscopy of Gram-stained smears of the muscle biopsies revealed no microbes; however, due to the greenish discoloration of the fascia and muscle, *Pseudomonas* spp. was suspected, and tobramycin, later replaced by ciprofloxacin, was added to the antibacterial regimen. Finally, MRI of the head detected ischemic strokes in the right corona radiate and the left cerebellum, possibly caused by septic emboli, but transthoracic echocardiography showed normal cardiac function and no valve vegetation.

**Figure 3:**
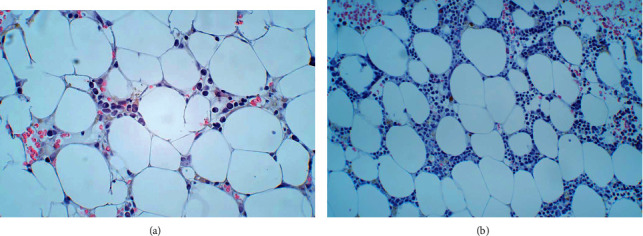
(a) Trephine biopsy showing markedly hypocellular bone marrow, with 5–10% cellularity. (b) Trephine biopsy showing a significant increase of bone marrow cellularity after treatment with IST.

The trephine biopsy showed hypocellular bone marrow (only 5–10% cellularity, Figure 3(a)) with only a small number of lymphocytes and plasma cells, and complete absence of both megakaryocytes, myelopoiesis, and erythropoiesis. The number of blast cells was not increased, and there were no signs of marrow fibrosis or gelatinous transformation of the bone marrow. The chromosome analysis showed normal male karyotype. Next-generation sequencing genetic testing, covering over 300 different genes, including genes involving in hereditary bone marrow failure syndromes, was without any specific finding. These findings were consistent with aplastic anaemia, but a mitomycin C test was not possible due to the low lymphocyte level. Eltrombopaq treatment was therefore started, and the G-CSF dose was also doubled, but granulocyte transfusions were not available.

Histological examination of muscle biopsies showed colonies of Gram-positive cocci and extensive muscle necrosis. All bacterial cultures of the muscle biopsies were negative, but 16S-ribosomal RNA (16s-rRNA) gene sequencing performed on bacterial DNA from muscle biopsies identified *Streptococcus dysgalactiae* (SD) in 3 out of 4 samples. Thus, the diagnosis streptococcal necrotizing myositis was confirmed, and antimicrobial treatment with meropenem and clindamycin without ciprofloxacin was continued.

The clinical picture was surprisingly peaceful despite the severe NSTI with widespread necrosis. The patient had intermittent low-grade fever, the erythema regressed, and the CRP level decreased to 100 mg/L. NSTI is usually regarded as a surgical emergency where early and aggressive surgical debridement of the infected muscle and fascia is imperative to secure survival. This usually requires multiple surgeries with removal of the affected muscles and fascia followed by a prolonged period of wound management before closure of the skin is possible. During this time period, adequate neutrophil and platelet function and counts are required to avoid superinfections and secure wound healing.

Severe AA requires treatment with allogenic stem cell transplantation or immunosuppressive therapy to improve the prognosis. AA left untreated incurs a high risk of death from infections. Since the clinical situation was stable, further surgery was deferred and intensive immunosuppressive therapy with horse antithymocyte globulin (ATG) and cyclosporine A combined with eltrombopaq was initiated. This had to be combined with ongoing antimicrobial therapy until neutrophil recovery was achieved. A summary of important clinical aspect is given in Table 1.

The patient was discharged from hospital after 3 months and was followed up weekly through outpatient care. Peripheral blood evaluation at discharge showed leucocytes 1.7 × 10^9^/L, neutrophils 0.6 × 10^9^/L, haemoglobin of 7.8 g/dL, reticulocytes 0.039 × 10^12^/L, and a thrombocyte count of 14 × 10^9^/L. A new bone marrow biopsy showed a significant increase of the cellularity with detectable haematopoiesis as signs of regeneration (Figure 3(b)).

## 3. Discussion

NSTIs are rapidly progressive infections associated with significant tissue destruction, systemic toxicity, and a high mortality rate if not treated early [9]. Timely identification and treatment including prompt surgery with extensive debridement are essential to secure survival [10, 11]. The current case presents a patient with severe multifocal NSTI as the initial presentation of severe AA; despite circulatory collapse, he stabilized after antibiotic and intensive care treatment and recovered without surgical debridement.

The patient fulfilled the generally accepted criteria for severe AA and had peripheral pancytopenia and bone marrow hypocellularity; the 1-year mortality rate if this disease is left untreated exceeds 80% and is mainly due to bacterial and fungal infections [12–15]. AA requires prompt treatment with either allogeneic stem cell transplantation (HSCT) or immunosuppressive therapy (IST) with ATG and cyclosporine to establish bone marrow function. Treatment in AA is individualized based on age, donor availability, comorbidities, and risk of infections. In the current case, standard IST in combination with eltrombopaq was the chosen first-line therapy, since there was no donor available, and the ongoing severe infection made him unfit for HSCT at the time of initiating the treatment.

The microbial aetiology of NSTIs can be polymicrobial or monomicrobial. Monomicrobial infections are predominantly caused by group A streptococcus (GAS) or other beta-haemolytic streptococci [16, 17]. The aetiology in our case was identified by 16S-rRNA gene sequencing that cannot distinguish between the two subspecies of *Streptococcus dysgalactiae* (SD). However, *Streptococcus dysgalactiae* subspecies *dysgalactiae* (SDSD) is almost exclusively a zoonotic pathogen [18], which makes *Streptococcus dysgalactiae* subspecies *equisimilis* (SDSE) the most likely cause of infection in our patient.

Reported risk factors for SDSE NSTIs are blunt trauma, age, male gender, and immune defects [1, 6, 7, 17, 19–21] that were all present in our patient. Furthermore, the patient's regular use of nonsteroidal anti-inflammatory drugs (NSAIDs) may have suppressed neutrophil functions and thereby masked early clinical signs and contributed to delayed diagnosis of NSTI [22, 23].

Neither clinical signs, radiological, nor laboratory findings can distinguish NSTIs from nonnecrotizing skin infections, and explorative surgery is required to establish the diagnosis [24]. Severe and localized pain is often the only early sign of infection in immunocompromised patients, but they may even experience less pain due to the inadequate immune response [25–27]. Hence, the diagnosis of NSTI can easily be overlooked and surgical exploration delayed [25]. This was also the case for our patient. Neutrophil migration to the site of infection is a crucial step in host defence [28]; the neutropenia may then have limited the endothelial injury and the overall systemic inflammatory response [29, 30] and thereby contributed to the successful outcome.

Streptococcal virulence factors are well-described contributors to the pathogenesis of NSTIs [10, 31, 32]. Putative virulence factors for SDSE include adhesins, toxins, and factors important for dissemination in human tissues and interference with the host immune responses. An example of such virulence factor is extracellular nuclease and streptodornase (DNase), which allows the bacteria to escape killing in neutrophil extracellular traps (NETs) [16, 33–37]. The extensive host inflammatory response to streptococcal infections, and especially neutrophil migration and degranulation, is considered to play a major role in tissue damage and systemic toxicity in these infections [4, 28]. Analysis of streptococcal NSTI patient tissue biopsies showed a strong correlation between bacterial load, neutrophil influx and degranulation markers, and tissue inflammation [38]. The mechanism behind neutrophil activation is incompletely understood, but streptococcal M-proteins have been shown to trigger activation and degranulation of neutrophils which contribute to vascular leakage and acute lung injury in the course of NSTI. Furthermore, streptococcal exotoxins may act as superantigens (SAgs) that can induce a broad and uncontrolled release of various cytokines [35]. Both tissue and serum measurements of cytokines correlate with clinical outcomes such as disease severity and 30-day mortality [38]. However, there is a significant interindividual variation in cytokine response after SAgs stimulation, and this observation is believed to be partially responsible for the heterogeneous clinical outcomes in patients with invasive GAS infections [39].

Several factors may have influenced the outcome in the current case. First, SDSE has fewer and less potent virulence factors compared with GAS. Second, neutropenia may have dampened the initial inflammatory response and been protective against pulmonary injury, so the patient avoided respiratory support and could be managed adequately with supportive care. Third, several studies have shown that T cell subsets are expanded and functionally abnormal in AA with abnormal response to immunological stimuli [40]. This might also have dampened both the early and late immunological responses.

Data on mortality and prognostic factors in NSTIs caused by SDSE are scarce. A 15-year retrospective survey in Western Norway found the 30-day mortality rate in SDSE NSTIs to be 31% [17]. Studies comparing invasive GAS and SD disease in general indicate that death among SD cases is more often related to age and comorbidity and more rarely to septic shock than in GAS infections [20, 41]. Bacteraemia is an independent predictor of mortality for NSTIs [42]. In the present case, repeated blood cultures were negative. Positive blood cultures are found in 40–60% of streptococcal NSTIs, and bacteraemia is more common in GAS NSTIs than in SDSE NSTIs [6, 43–45]. Although tissue biopsies showed Gram-positive cocci in the necrotic muscle, there was no growth in the tissue or fluid cultures. This was most likely due to the ongoing antibiotic therapy.

Early initiation of antibiotic treatment can limit the expansion of the total bacteria load and thereby improve prognosis [46]. Empirical antibiotic treatment of NSTIs is often inadequate. In a UK study, 92% of patients with severity class IV skin and soft tissue infections, including NSTIs, received antibiotics considered inappropriate compared to UK guidelines [47]. However, our patient received adequate broad-spectrum antibiotics (i.e., meropenem plus clindamycin) consistent with the recommendations for treatment of streptococcal beta-haemolytic NSTIs from admission [11]. Treatment including clindamycin has shown to be superior to beta-lactam antibiotics in two observational studies on streptococcal NSTIs [48, 49] and has a crucial role in controlling local inflammation by reducing local toxin production [50].

To the best of our knowledge, this is the first case report to describe a patient who presents with NSTI as the initial presentation of AA that successfully could be treated with IST. Ugarte-Torres et al. present a patient with relapsed AA that developed bilateral lower extremity necrotizing fasciitis caused by multidrug-resistant *Aeromonas hydrophilia*. As with our patient, IST with ATG and cyclosporine A was initiated, but the patient passed away after subsequent surgery [26].

## 4. Conclusion

The current case illustrates that the clinical course of NSTI in severely neutropenic patient can be atypical and easily overlooked. The clinical improvement despite severe neutropenia and stabilization during immunosuppressive therapy suggests that tissue damage in NSTI may in part be caused by secondary immunological factors.

## Figures and Tables

**Table 1:** Important aspect of the clinical course.

Timeline	Day 1	Day 3	Day 5	Day 7	Day 8	Day 17	Day 77	Day 90
	Transferred to ICU	Discharged from ICU						Discharged from hospital
C-reactive protein	592 mg/L					170 mg/L		1 mg/L
Antimicrobial therapy	Antibiotics initiated						Treatment discontinued	
CT scan or/and MRI	X		X					
Surgical exploration				X				
AA directed therapy					Eltrombopaq initiated	ATG and cyclosporine initiated		
Neutrophil count	0.1 × 10^9^/L					0.0 × 10^9^/L		0.6 × 10^9^/L

## Data Availability

The data generated or analyzed during this study are included within the article.

## Abbreviations

AA: Aplastic anaemia
GAS: Group A streptococcus
MRI: Magnetic resonance tomography imaging
NSTI: Necrotizing soft tissue infections
SDSE: *Streptococcus dysgalactiae* subspecies *equisimilis.*
